# Chemical characterization and antioxidative activity of four 3-hydroxyl-3-methylglutaroyl (HMG)-substituted flavonoid glycosides from *Graptopetalum paraguayense* E. Walther

**DOI:** 10.1186/s40529-015-0088-4

**Published:** 2015-04-21

**Authors:** Hsin-Yi Liu, Hsin-Yi Peng, Shih-Lan Hsu, Ting-Ting Jong, Su-Tze Chou

**Affiliations:** 1 Department of Chemistry, National Chung Hsing University, 250 Kuo-kuang Road, Taichung, 402 Taiwan; 2 Department of Food and Nutrition, Providence University, 200, Sec. 7, Taiwan Boulevard, Taichung, 43301 Shalu Dist Taiwan; 3 Department of Education and Research, Taichung Veterans General Hospital, 1650, Sec. 4, Taiwan Boulevard, Taichung City, 40705 Taiwan

**Keywords:** Antioxidative activity, Flavonoid glycoside, Graptopetalum paraguayense, 3-hydroxyl-3-methylglutaroyl (HMG)

## Abstract

**Background:**

*Graptopetalum paraguayense* E. Walther is a popular traditional Chinese herb and possesses several health benefits. In earlier studies, we demonstrated that *G. paraguayense* showed no genotoxicity and showed several biological activities. However, the constituents of *G. paraguayense* have not been studied yet. In this present study, we isolated and identified the constituents of the leaves of *G. paraguayense* E. Walther.

**Results:**

A total of seven flavonoid compounds were isolated from the methanolic extract of *G. paraguayense*. The four major compounds isolated were flavonoid glucoside derivatives of quercetin (**1**, **3**) and kampferol (**2**, **4**), each presenting a 3-hydroxyl-3-methylglutaroyl (HMG) substituent; compounds **3** and **4**—the **2**´´-acetyl derivatives of **1** and **2**, respectively—are novel compounds isolated from nature for the first time. High-performance liquid chromatography for the quantitative analyses of the four major HMG-substituted flavonoid glycosides in *G. paraguayense* E. Walther were accomplished to acquire the high yields of **1**–**4** in the methanolic extract (4.8, 5.7, 4.3, and 2.5 mg/g, respectively). Furthermore, the antioxidant activities, including radical-scavenging, reducing power and lipid peroxidation inhibitory effects of these isolated flavonoids were also evaluated. All seven of the isolated flavonoid compounds possessed antioxdative activity.

**Conclusions:**

In this study of the constituents of the leaves of *G. paraguayense* E. Walther, we isolated four major components from its methanolic extract and determined their structures to be (acetylated) HMG-substituted flavonol glycosides, which are rare in nature. All seven of the isolated compounds possessed antioxdative activity, and those flavonoid compounds may be responsible for the functional ingredients in *G. paraguayense*. Further investigation of their bioactivities or pharmacological activities will be continued.

**Electronic supplementary material:**

The online version of this article (doi:10.1186/s40529-015-0088-4) contains supplementary material, which is available to authorized users.

## Background

*Graptopetalum paraguayense* E. Walther is a popular traditional Chinese herb and possesses several health benefits. According to an archaic Chinese prescription, *G. paraguayense*, belonging to the *Crassulaceae* family, is used for alleviating hepatic disorders, lowering blood pressure, relieving pains and infections. We had shown that *G. paraguayense* showed no genotoxicity by using Ames test, *in vitro* chromosome aberration assay and *in vivo* erythrocyte micronucleus assay. Additionally, the acute oral toxicity and 28-day repeated feeding toxicity tests showed that the aqueous extract of *G. paraguayense* is generally considered to be safe, with an no observed adverse effect level (NOAEL) value of 1.0 g/kg BW in rats (Chou et al. [2005]; Chung et al. [2012]). In earlier studies, we demonstrated several biological activities of *G. paraguayense*, including its dose-dependent inhibition of mushroom tyrosinase-catalyzed oxidation of L-dopa (Huang et al. [2005]), radical-scavenging and lipid peroxidation-preventive effects (Chung et al. [2005]), and antihepatoma potential (Chen et al. [2008]). Further, a 50% aqueous ethanolic extract of *G. paraguayense* (GE50) acts as a mixed-type inhibitor of angiotensin-converting enzyme (ACE) (Chen et al. [2007]), and our *in vivo* study confirmed that daily oral administration of GE 50 (2.5 g/kg BW) for 4 weeks reduces blood pressure in spontaneously hypertensive rats (Chung et al. [2009]) and provide hepatoprotection against *tert*-butylhydroperoxide- and CCl_4_-induced oxidative liver damage in rats (Chou et al. [2008]). In an 8-week clinical study, Lin et al. noted that oxidative stress in hypercholesterolemic subjects decreases on daily consumption of 100 g *G. paraguayense* (Lin et al. [2011]). Duh et al. observed that an aqueous extract of *G. paraguayense* has hepatoprotective effects by promoting antioxidant and antiinflammatory activities in an animal model of carbon tetrachloride (CCl_4_)-induced liver damage (Duh et al. [2011]). A reported patent depicted that *G. paraguayense* is potent for the protection of animal liver diseases and medical conditions, such as inflammation, steatosis, and fibrosis. In particular, *G. paraguayense* inhibits proliferation of activated hepatic stellate cells, which play a pivotal role in liver fibrosis (Hsu [2006]). Furthermore*, G. paraguayense* is useful against fibrosis or inflammation of tissues or organs other than the liver, in particular lung, kidney, and bladder. They mentioned in the patent that clinical trial results indicate almost all patients (14 persons) completely recovered from hepatic steatosis, thus, *G. paraguayense* is effective in patients with fatty liver.

**Figure 1:**
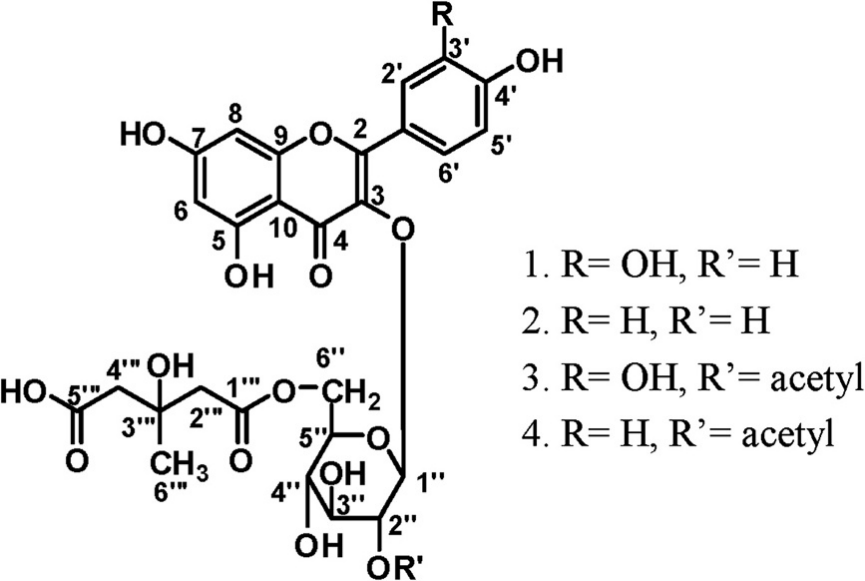
Structures of **1**–**4**.

However, the constituents of *G. paraguayense* have not been studied yet. In this present study, we isolated and identified several components of the herb and by using high-performance liquid chromatography (HPLC), we performed quantitative analysis for the four major compounds **1**–**4** (structures see Figure 1) which appeared to be the main components in the crude methanol extract. In addition, we used several assays to test the antioxidant potentials of these isolated compounds, including their radical-scavenging activities, reducing power, and antioxidative effects on Fe/ascorbate–induced lipid peroxidation in a liposome model system. Herein, we compare these results with those of quercetin, a reference antioxidant.

## Methods

### General experimental procedures

Optical rotations were measured using a Perkin–Elmer 241 polarimeter. IR spectra were recorded using a Perkin–Elmer Spectrum 100 FT-IR spectrometer. UV spectra were recorded using a Cintra UV 101 spectrometer.^1^H NMR spectra were recorded using Varian Unity Inova 400 and 600 MHz FT-NMR spectrometers; chemical shifts (δ) are expressed herein in units of ppm, with coupling constants (*J*) in Hz. HR-FAB/MS spectra were recorded using a Finnigan Thermo Quest MAT 95XL mass spectrometer. Column chromatography (CC) was performed using silica gel 60 F_254_ (70–230 and 230–400 mesh, Merck), an ODS column [Lichroperp RP-18 (15–25 μm, Merck)], and Sephadex LH-20 (Amersham Biosciences, Sweden). Thin layer chromatography (TLC) and preparative TLCs were performed using precoated silica gel plates; spots were visualized at 254 and 365 nm. Preparative HPLC was performed using an Agilent 1100 series system coupled with a UV detector.

### Plant material

The plant, *G. paraguayense*, was identified by an herb expert, Mr. Chiu Nien-Yuan, Department of Pharmacology, China Medical University (Taiwan). The leaves of *G. paraguayense* were collected from a farm in Yunlin County in Taiwan. A few batches of leaves samples were bought and found consistent by their HPLC profiles.

### Extraction and isolation

The freeze-dried leaves of *G. paraguayense* (GP, 530 g) were extracted with n-hexane; the residue was extracted with methanol and then this extract was concentrated. The methanolic extract (89.6 g) of *G. paraguayense* was partitioned between EtOAc and water, the aqueous layer discarded, and the organic layer dried over Na_2_SO_4_. After evaporation of the solvent under reduced pressure, we obtained 10.0 g of EtOAc extract (GP-EtOAc, yield from methanolic extract). The GP-EtOAc extract was coated with silica gel and subjected to CC (SiO_2_, 230–400 mesh, 9.5 × 22 cm), eluting with a gradient of hexane, EtOAc, and MeOH (hexane/EtOAc, 9:1, 2:8, 4:6, 8:2, 0:1; then EtOAc/MeOH, 3:1, 0:1) to yield seven fractions. Fraction 6 was subjected to ODS CC (Lichroperp RP-C18, 15–25 μm, Merck), eluting with a gradient of MeOH and H_2_O (MeOH/H_2_O, 8:2, 7:3, 1:1) to yield three fractions. Fraction 3 (MeOH/H_2_O, 1:1) was further purified six times through ODS CC (gradient from MeOH/H_2_O to MeOH) to obtain compounds **1** (30 mg), **2** (46 mg), **3** (51 mg), and **4** (35 mg).

### Experimental data of the novel flavonoid glycosides (**3**) and (**4**)

Quercetin 3-*O*-[6´´-(3-hydroxyl-3-methylglutaroyl)]-[2´´-acetyl]-β-D-glucopyranoside (**3**): C_29_H_30_O_17_; [α]D^24^ = −46.6° (c = 0.16, MeOH); UV (MeOH) λ_max_(log ε): 208 (3.17), 257 (2.81), 354 (2.76) nm; IR ν_max_ (MeOH) cm^−1^: 1764; (+) ESI-MS m/z: 673 [M + Na]^+^, 651 [M + H]^+^ ; (+) ESI-MS^2^ m/z: 349 [M + H – quercetin]^+^, 303 [quercetin + H]^+^; HR(+)ESI/MS [M + H]^+^m/z, 651.1587 (calcd for C_29_H_30_O_17_, 651.1556). ^1^H NMR (d_6_-acetone, 600 MHz) and ^13^C NMR (d_6_-acetone, 150 MHz) spectral data of **3** are provided in Table 1. Kampferol 3-*O*-[6´´-(3-hydroxyl-3-methylglutaroyl)]-[2´´-acetyl]*-* β-D- glucopyranoside (**4**): C_29_H_30_O_16_; [α]D^24^ = −43.0° (c = 0.40, MeOH); UV (MeOH) λ_max_ (logε): 209 (3.18), 266 (3.00), 350 (2.94) nm; IR ν_max_ (MeOH) cm^−1^: 1764; (+)ESI-MS m/z: 657 [M + Na]^+^, 635 [M + H]^+^; (+)ESI-MS2 m/z: 349 [M + H – kaempferol]^+^, 287 [kaempferol + H]^+^; HR(+)ESI/MS: [M + H]^+^ m/z 635.1632 (calcd for C_29_H_3_0O_17_, 635.1607). ^1^H NMR (d_6_-acetone, 600 MHz) and ^13^C NMR (d_6_-acetone, 150 MHz) spectral data of 4 are provided in Table 1.

**Table 1:** **Summarizes pertinent** ^**1**^ **H and** ^**13**^ **C NMR spectral data and assignments**

Position	Compound 1		Compound 2		Compound 3		Compound 4	
	(in Acetone-***d***_6_)		(in Acetone-***d***_6_)		(in Acetone-***d***_6_)		(in Acetone-***d***_6_)	
	**δ**_**H**_	**δ**_**C**_	**δ**_**H**_	**δ**_**C**_	**δ**_**H**_	**δ**_**C**_	**δ**_**H**_	**δ**_**C**_
**2**		158.4		158.6		158.0		158.1
**3**		135.2		133.3		134.4		134.2
**4**		178.9		177.4		178.5		178.6
**5**		162.6		161.2		162.8		162.8
**6**	6.28 (1H, d, 1.8)	99.5	6.28 (1H, d, 1.8)	98.8	6.26 (1H, d, 1.8)	99.4	6.26 (1H, d, 1.8)	98.2
**7**		165.1		164.2		164.8		164.8
**8**	6.52 (1H, d, 1.8)	94.6	6.52 (1H, d, 1.8)	93.7	6.49 (1H, d, 1.8)	94.4	6.50 (1H, d, 1.8)	93.5
**9**		157.8		156.5		157.8		157.8
**10**		105.4		104.1		105.5		103.1
**1’**		122.7		120.9		122.8		121.7
**2’**	7.77(1H, d, 1.8)	117.2	8.12 (1H, d, 9.0)	130.7	7.76(1H, d, 2.4)	117.1	8.08 (1H, d, 9.0)	132.0
**3’**		145.2	6.96 (1H, d, 8.4)	115.1		145.2	6.97 (1H, d, 9.0)	115.4
**4’**		149.1		160.0		148.9		160.8
**5’**	6.94 (1H, d, 8.4)	115.6	6.96 (1H, d, 8.4	115.1	6.95 (1H, d, 8.4)	115.7	6.97 (1H, d, 9.0)	115.4
**6’**	7.68 (1H, dd, 1.8,8.4)	123.1	8.12 (1H, d, 9.0)	130.7	7.63 (1H, dd, 2.4,8.4)	123.0	8.08 (1H, d, 9.0)	132.0
**1”**	5.30 (1H, d, 7.2)	104.2	5.28 (1H, d, 7.8)	101.3	5.61 (1H, d, 7.8)	100.2	5.62 (1H, d, 7.8)	100.1
**2”**	3.40-3.52 (1H, m)	75.2	3.45 (1H, dd, 7.8, 9.0)	74.1	5.01 (1H, dd, 7.8, 9.6)	75.0	4.97 (1H, dd, 8.4, 9.6)	74.8
**3”**	3.40-3.52 (1H, m)	77.7	3.40 (1H, t, 9.0)	76.4	3.70 (1H, t, 9.0)	75.3	3.68 (1H, t, 9.0)	75.2
**4”**	3.40-3.52 (1H, m)	70.6	3.48-3.54 (1H, m)	70.1	3.42 (1H, dd, 9.0, 9.6)	71.0	3.46 (1H, dd, 9.0, 9.6)	71.2
**5”**	3.40-3.52 (1H, m)	75.1	3.48-3.54 (1H, m)	74.1	3.55 (1H, dd, 1.8, 7.8)	74.9	3.54 (1H, dd, 1.8, 7.8)	75.0
**6”**	4.11 (1H, dd, 5.4, 12.0)	64.0	4.10 (1H, dd, 6.0, 12.0)	63.2	4.12 (1H, dd, 5.4, 12.0)	63.7	4.10 (1H, dd, 5.4, 12.0)	63.8
	4.22 (1H, dd, 1.8, 12.0)		4.22 (1H, dd, 1.8, 12.0)		4.24 (1H, dd, 1.8, 12.0)		4.22 (1H, dd, 1.8, 12.0)	
**1”’**		171.6		170.0		171.5		171.4
**2”’**	2.52 (1H, d, 14.4, H-2”’A or H-2”’B)	45.7	2.51 (1H, d, 14.4, H-2”’A or H-2”’B)	45.7	2.51 (1H, d, 13.8, H-2”’A or H-2”’B)	45.7	2.48 (1H, d, 13.8, H-2”’A or H-2”’B)	45.7
	2.56 (1H, d, 14.4, H-2”’A or H-2”’B )		2.56 (1H, d, 14.4, H-2”’A or H-2”’B )		2.54 (1H, d, 13.8, H-2”’A or H-2”’B )		2.51 (1H, d, 13.8, H-2”’A or H-2”’B )	
**3”’**		69.9		68.7		69.9		69.9
**4”’**	2.50 (1H, d, 15.6, H-4‘”A or H-4”’B)	45.1	2.52 (1H, d, 15.6, H-4”’A or H-4”’B)	44.9	2.48 (1H, d,15.6, H-4”’A or H-4”’B)	45.1	2.47 (1H, d, 15.6, H-4”’A or H-4”’B)	45.0
	2.59 (1H, d, 15.6, H-4”’A or H-4”’B)		2.59 (1H, d, 15.6, H-4”’A or H-4”’B)		2.56 (1H, d, 15.6, H-4”’A or H-4”’B)		2.54 (1H, d, 15.6, H-4”’A or H-4”’B)	
**5”’**		173.6		172.2		173.5		173.4
**6”’**	1.23 (3H, s)	27.5	1.24(3H, s)	27.0	1.20 (3H, s)	27.5	1.19 (3H, s)	27.4
**1””**						170.5		170.3
**2””**					2.10 (3H, s)	21.1	2.10 (3H, s)	21.0

### Quantitative analysis through HPLC

Calibration solutions of standards **1**–**4** were prepared at concentrations of 2–500 μg/mL. The analysis was performed using a reversed-phase Lichrospher 100 RP-18 column (250 mm × 4.6 mm, 5 μm). The extracts were separated using aqueous solutions containing 0.1% phosphoric acid (A) and acetonitrile (B). The eluent flow rate was maintained at 1 mL/min; the injection volume was 20 μL; the detection wavelength was 270 nm; the column temperature was set at ambient temperature. The elution program with a linear gradient was optimized as follows: 0 min, 6% B; 30 min, 45% B; 33 min, 100% B; 40 min, 6% B, followed by a 5-min conditioning period. The identities of the analytes were confirmed by comparing their HPLC retention times with that of the analytical standard. Calibration was achieved through linear regression analysis performed at eight different concentrations (2, 10, 25, 50, 100, 200, 400, 500 μg/mL). Quantitative analysis was performed after integration of the peaks. All experiments in this study were carried out in triplicate.

### Antioxidative activity assay: DPPH radical-scavenging activity

The DPPH radical-scavenging activities of the compounds isolated from *G. paraguayens* were measured using a method described previously (Chen et al. [2008]). An aliquot of an extracted compound or quercetin (0.1 mL, 0.005–600.0 μM) was mixed with 100 mM Tris–HCl buffer (pH 7.4, 0.4 mL) and then added to 500 μM DPPH in EtOH (0.5 mL; to a final concentration of 250 μM). The mixture was shaken vigorously and left in the dark at room temperature for 20 min. The absorbance of the mixture was measured spectrophotometrically at 517 nm. The ability to scavenge DPPH radicals was calculated using the equation: scavenging effect (%) = [1 – (absorbance of sample at 517 nm/absorbance of control at 517 nm)] × 100%. The value of IC_50_, the effective concentration at which the antioxidant activity was 50%, was obtained by interpolation from linear regression analysis. All assays were conducted at least three times with three different sample preparations. All data are expressed as the mean ± standard deviation (SD).

### Antioxidative activity assay: ABTS^+^ radical-scavenging activity

The ABTS^+^ radical-scavenging activities of the isolated compounds were determined through ABTS decolorization assays (Chen et al. [2008]). The ABTS^+^ radical generation system, comprising 2.45 mM potassium persulfate (K_2_S_2_O_8_) and 7 mM ABTS salt in 0.01 M phosphate-buffered saline (pH 7.4), was maintained for 16 h at room temperature in the dark. Each tested compound (0.01–600.0 μM) was diluted (100X) with the ABTS^+^ solution to a total volume of 1 mL and then left to react for 10 min. The control (without sample) was used as a blank; 990 μL of PBS was added to these control samples instead. All assays were conducted at least three times with three different sample preparations. All data are expressed as the mean ± standard deviation (SD).

### Antioxidative activity assay: antioxidative effect on liposome peroxidation

Liposome peroxidation was induced by Fe^2+^-ascorbate and aquatinted by the malondialdehyde-thiobarbituric acid (MDA-TBA) adduct, according to the method described by Liao and Yin but with slight modification (Liao and Yin [2000]). Liposomes (multi-lamellar vesicles) were prepared from phosphatidyl choline (PC) (30 mg), cholesterol (12 mg), and dicetyl phosphate (3 mg) at 4°C. The solvents (CHCl_3_, MeOH) used for liposome preparation were removed and a lipid film was formed through rotary evaporation under a N_2_ flush. The liposomes were then suspended in 50 mM sodium phosphate buffer (pH 7.2, 10 mL). A mixture of the liposome suspension (1 mL), sodium phosphate buffer (0.6 mL), 25 mM FeCl_3_ (0.05 mL), 25 mM ascorbic acid (0.05 mL), and the isolated compounds (0.08–600.0 μM, 0.25 mL) was incubated for 1 h at 37°C. After incubation, the solution was mixed with TBA (0.4% in 0.2 M HCl) and BHT (0.2% in 95% EtOH) at a ratio of 1:2:0.3 and then heated at 100°C for 20 min. After cooling, an equal volume of n-BuOH was added to the mixture to extract the chromogen. The absorbance of the n-BuOH layer was measured spectrophotometrically at 532 nm. The ability to inhibit MDA formation was calculated using the equation: inhibition effect (%) = [1 – (absorbance of sample at 532 nm/absorbance of control at 532 nm)] × 100%. The MDA level for no compound added was 18.04 μM in the control. The standard reference compound for all antioxidative tests was quercetin. All assays were conducted at least three times with three different sample preparations. All data are expressed as the mean ± standard deviation (SD).

### Antioxidative activity assay: reducing power

The reducing powers of the *G. paraguayense* extracts and the isolated compounds were determined according to the method of Yen and Chen ([1995]). An isolated compound (0.001–1.0 μM) was mixed with an equal volume of 0.2 M phosphate buffer (pH 6.6) and 1% potassium ferricyanide and then incubated at 50°C for 20 min. An equal volume of 1% trichloroacetic acid was added to stop the reaction and then the mixture was centrifuged (2790 g, 10 min). The supernatant was mixed with distilled water and 0.1% FeCl_3_ at a ratio of 1:1:0.2 and then the absorbance was measured at 700 nm. All assays were conducted at least three times with three different sample preparations. All data are expressed as the mean ± standard deviation (SD).

## Results and discussion

**Figure 2:**
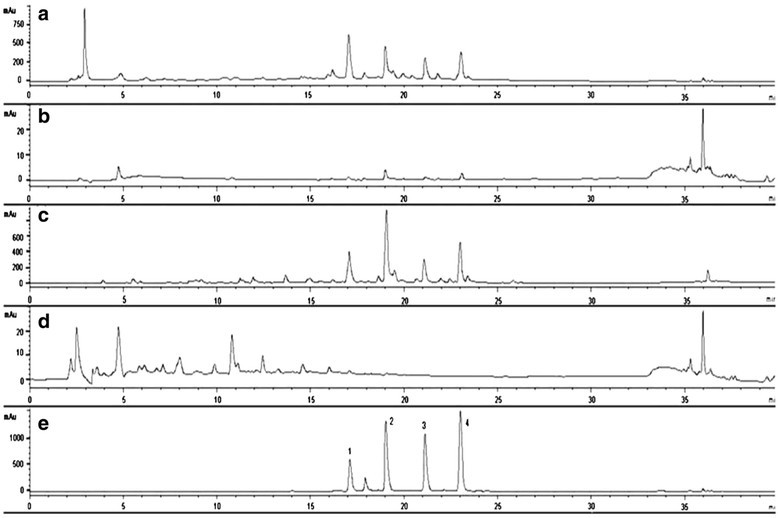
HPLC chromatograms (270 nm) of the **(a)** crude MeOH extract of *G. paraguayense*, **(b)** *n*-hexane extract, **(c)** EtOAc extract, **(d)** water extract, and **(e)** isolated flavonoid glycosides **1**–**4** (each 250 ppm). (Note: The impurity appearing at R_f_ 18 min on **(e)** is due to the contamination of compound **6**.

Figure 2 displayed the HPLC traces of the MeOH extract and the three partitioned fractions (GP-Hex, GP-EtOAc, GP-H_2_O) of the MeOH extract from the leaves of *G. paraguayense*. Since the GP-EtOAc fraction contained the same major four compounds of the herb as found in the crude MeOH extract, we subjected the EtOAc fraction to further purification. After successive normal and reversed-phase chromatography, we obtained a total of seven flavonoid compounds. The four major components isolated were quercetin 3-*O*-[6´´-(3-hydroxyl-3-methylglutaroyl)]-β-d- glucopyranoside (**1**), kampferol 3-*O*-[6´´-(3-hydroxyl-3-methylglutaroyl)]-β-d- glucopyranoside (**2**), quercetin 3-*O*-[6´´-(3-hydroxyl-3-methylglutaroyl)-2´´-acetyl]- β-d-glucopyranoside (**3**), and kampferol 3-*O*-[6´´-(3-hydroxyl-3-methylglutaroyl)- 2´´-acetyl]-β-d- glucopyranoside (**4**); the three minor components were isoquercetin (**5**), kaempferol 3-*O*-β-d-glucopyranoside (**6**), and kaempferol (**7**). Among these components, compounds **1**, **2**, and **5**–**7** were known; compounds **3** and **4**–derivatives of **1** and **2**, respectively—are novel compounds isolated from nature for the first time in this study. Compounds **3** and **4** are new acylated derivatives of **1** and **2**, respectively. The characteristic feature of these major flavonoid glycosides is the 3-hydroxyl-3- methylglutaroyl (HMG) substituent on their sugar moieties. Compound 1 had been isolated previously as a UV shield from Himalayan Rheum species and from another Euphorbia species (Iwashina et al. [2004]; Liu et al. [2004]), compound **2** had been found solely in 10-year-old callus cultures of Mexican lime, with the authors of that study suggesting that the compound may have been stress-induced and found only in the wounded tissues from which the callus was derived (Berhow et al. [1994]). We used ESI and FAB mass spectrometry and 1D and 2D NMR spectroscopy to elucidate the structures of the four HMG-substituted flavonoid glycosides **1**–**4**.

### Structural elucidation of **1**–**4**

The characteristic UV absorptions at 256 and 357 nm of compounds **1**–**4** and the presence of signals for 15 aromatic carbon atoms in the downfield region in their ^13^C NMR spectra indicated their flavonoid skeleton structures. We deduced that the A-ring of each of the flavonoids **1**–**4** featured a 5,7-disubsituted structure, due to the appearance of a small coupling constant (2 Hz) between H-6 and H-8 in the ^1^H NMR spectrum; the B-ring featured either the ABX (i.e., 1 and 3) or A_2_X_2_ (i.e., **2** and **4**) system of protons that is characteristic of quercetin or kaempferol, respectively. For each of these compounds, we confirmed that the sugar moiety was a glucose unit, as revealed by the relationships among the coupled protons in the ^1^H-2D COSY spectra and by the chemical shifts of the corresponding signals of six carbon atoms-for example, at 104.2 (C-1´´), 75.2 (C-2´´), 77.7 (C-3´´), 70.6 (C-4´´), 75.0 (C-5´´), and 64.0 (C-6´´) ppm for compound **1** - in ^13^C NMR spectra. The β-glycoside linkage was established on the large coupling constant (7.8 Hz) of the glucose proton H-1´´. We established the site of attachment of the glucose moiety to the flavonoid as being the C-3 position, due to the correlation found between proton H-1´´ and C-3 in the HMBC spectra.

In addition to the signals in the ^1^H and ^13^C NMR spectra for the glucose moieties in these compounds, the remaining signals including an upfield (ca. 2.45-2.55 ppm) multiplet corresponding to four protons, a signal at ~1.2 ppm for methyl protons, and peaks for six carbon atoms-two C = O carbon atoms (171.6, 173.6 ppm); one oxygenated quaternary carbon atom (69.9 ppm), two upfield methylene carbon atoms (45.1, 45.7 ppm), and one methyl carbon (27.5 ppm)-that characterized the HMG substituent on these flavonoid glycosides. We confirmed that the attachment of the HMG units to the sugar moieties occurred at the C-6 position of the glucose unit, through HMBC correlation of the H-6´´ protons of the glucose with the carbonyl carbon (C = O) of the HMG unit.

For compounds **3** and **4**, spectroscopic analysis revealed one extra substituent, which corresponded to an acetyl group (i.e. a methyl proton signal at 2.1 ppm and a carbonyl carbon signal at 170.5 ppm for compound **3**). From the correlations between the glucose H-2´´ protons and the acetyl C = O carbon atoms in the HMBC spectra, we deduced that the site of attachment was the C-2´´ hydroxyl group.

### Quantitative analyses of the amounts of the flavonoids in the leaves

Figure 2a reveals that The MeOH extract of the freeze-dried leaves of *G. paraguayense* powder yielded the four major compounds **1**–**4**. According to HPLC analysis, with UV detection at 270 nm, the retention times of compounds **1**–**4** were 17.0, 19.0, 21.1, and 23.0 min, respectively. Figure 2e displays the chromatogram of the crude extract sample spiked with the four isolated standards; the signals of the major components were coherent.

Through quantitative analysis based on triplicate injections into the HPLC system, we found that the linearity of detection of the four compounds ranged from 2 to 500 ppm (μg/mL), with the squared correlation coefficient exceeding 0.999 in each case (Table 2). These experiments provided the necessary information for estimating the limits of detection (LODs) from the slopes of the calibration curves and the threefold standard deviation (3S) of the lowest detectable concentration. We estimated the LODs for compounds **1**–**4** to be 0.6, 0.8, 0.5, and 0.4 μg/mL, respectively.

**Table 2:** **Linear ranges, calibration curves, correlation coefficients, and detection limits for 1–4 analyzed using HPLC**

Compound	Linear range (μg /mL)	Linear equation	Squared correlation coefficient (***r***^2^)	LOD* (μg/mL)
**1**	2–500	*y* = 20.930x–20.053	0.9999	0.6
**2**	4–500	*y* = 14.028x–0.100	0.9998	0.8
**3**	2–500	*y* = 25.918x–24.031	0.9998	0.5
**4**	2–500	*y* = 29.500x–16.592	0.9999	0.4

*LOD = 3 × SD/slope.

The overall yields of the four flavonoids **1**–**4** from the crude MeOH extract of the leaves of G. *paraguayense* were 4.8, 5.7, 4.3, and 2.5 mg/g, respectively, totaling 17.3 mg/g of the extract. This content corresponds to 5.0 mg/g of the freeze-dried sample and further to 0.2 mg/g of the fresh leaves, after taking into account a measured water content of 97%. It indicated that a high abundance of these special flavonoids in the leaves of this plant.

### Antioxidative activities of the seven compounds isolated from *G. Paraguayense*

**Figure 3:**
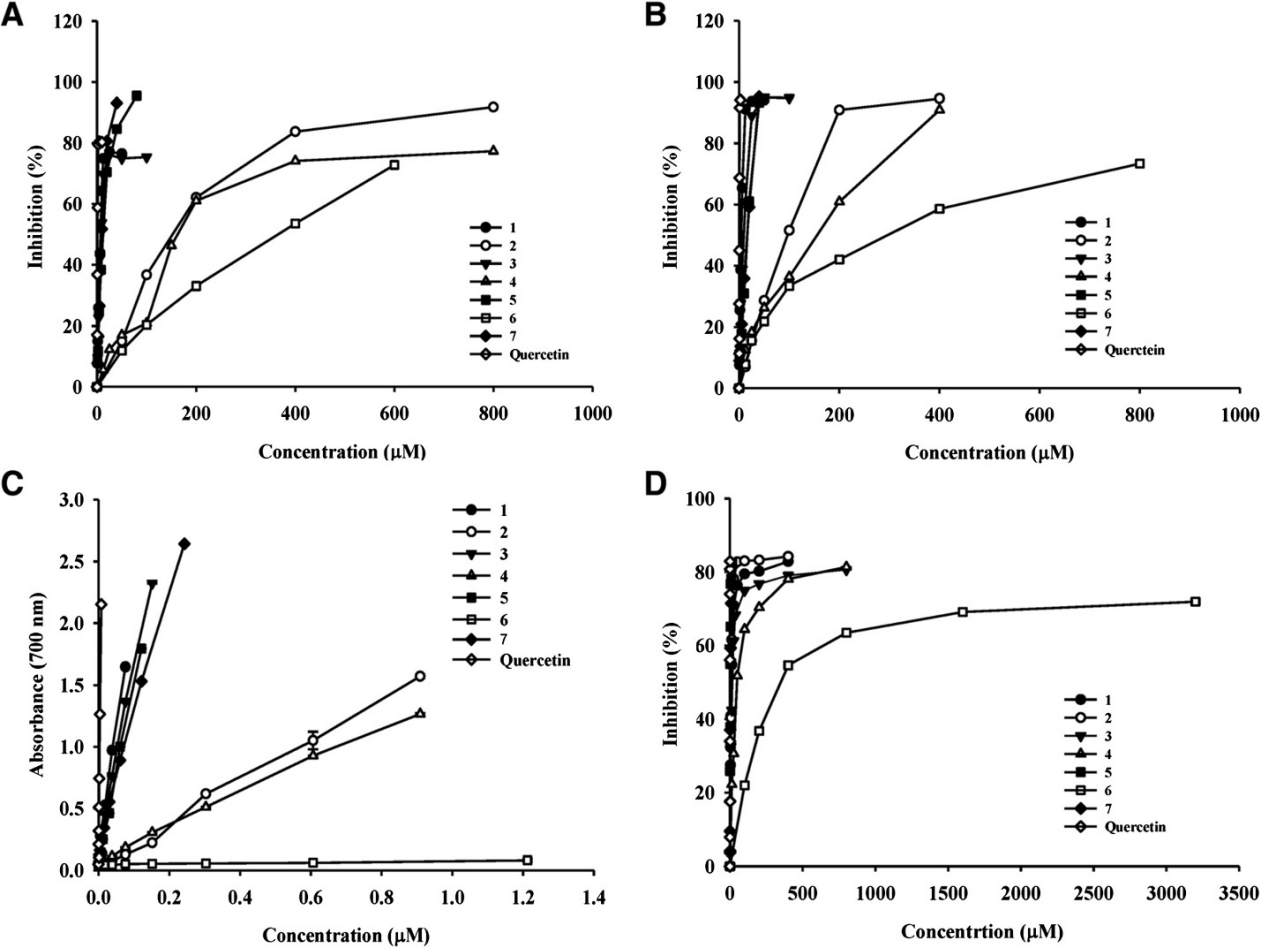
Scavenging effects of the compounds **1**–**7** isolated from *G. paraguayens* on **(A)** DPPH, **(B)** ABTS radical, **(C)** reducing power, and **(D)** Fe^2+^/ascorbate-induced lipid peroxidation. Each value represents mean ± SD (n = 3). **1**, quercetin 3-*O*-(6´´-HMG) -β-d-glucopyranoside; **2**, kaempferol 3-*O*-(6´´-HMG) -β-d-glucopyranoside; **3**,quercetin 3-*O*-(2´´-acetyl)*,*(6´´-HMG) -β-d-glucopyranoside; **4**, kaempferol 3-*O*-(2´´-acetyl),(6´´-HMG)-β-d-gluco-pyranoside; **5**, quercetin 3-*O*-β-glucoside; **6**, kaempferol 3-*O*-β-glucoside; **7**, kaempferol.

In this present study, we evaluated the antioxidative activities-namely the radical-scavenging activities, reducing powers and anti-lipid peroxidation activities-of the flavonoids isolated from *G. paraguayense*. Many food materials possess free radical-scavenging ability and their effect on radical scavenging was thought to be due to their hydrogen-donating ability (Yamaguchi et al. [1998]). Figures 3A - B show the dose–response curves of DPPH and ABTS radical-scavenging activities of the flavonoids isolated from *G. paraguayense*. It was found that the radical-scavenging abilities of all isolated compounds increased with the increase in their concentrations. Figure 3C revealed the reducing powers-that is, the abilities of the isolated compounds to reduce Fe^3+^ to Fe^2+^. The reducing powers of isolated compounds also increased with the increase of concentrations. The results revealed that the isolated flavonoids displayed reductive potential and could serve as an electron donor and terminate the radical chain reaction (Yen and Chen [1995]). Lipid peroxidation is a typical free radical oxidation and proceeds via a cyclic chain reaction. Liposomes, artifical biomembanes, have been used extensively as a model system for *in vitro* lipid peroxidation studies (Duh et al. [1999]; Yin and Cheng [1998]; Tsuda et al. [1996]). The antioxidant activities of the isolated compounds on MDA formation in Fe^2+^/ascorbate-mediated lipid peroxidation in the liposome system were shown in Figure 3D. It was also found that the inhibitory effect on the lipid peroxidation of isolated compounds was in a concentration-dependent manner. The mechanism of the inhibitory effect through which the isolated compounds protect against lipid peroxidation may involve radical scavenging and reducing capability. Many studies have showed that flavonoids not only have anti-radicals activities but also have the transition metal ions-chelating properties which may afford protection against lipid peroxidation (Cai et al. [2006]; Malešev and Kuntić [2007]; Olennikov et al. [2014]). Lipid peroxidation is an oxidative alteration of polyunsaturated fatty acids in cell membranes that leads to cellular damage, which is strongly associated with, for example, aging and carcinogenesis (Yagi [1987]). In addition, lipid peroxidation is a major deteriorative reaction in the processing and storage of lipid-containing foods (Duthie [1993]). Hence, our findings confirm the health-promoting potential of *G. paraguayense*.

Table 3 lists the half-inhibition concentrations (IC_50_) for the radical-scavenging capacities and anti-lipid peroxidation activities. In terms of their IC_50_ values, the radical-scavenging activities and reducing powers of the isolated compounds decreased in the order **1** > **3** > **5** > **7** > **2** > **4** > **6**; in contrast, the anti-lipid peroxidation capacities followed the order **5** > **7** > **2** > **1** > **4** > **3** > **6**. Based on these results, the radical-scavenging capacities of the glycosides are lower than that of their aglycones. In addition, the acetylated HMG-substituted flavonoid glycosides **3** and **4** possessed weaker antioxidative activities than those of **1** and **2** (Table 3 and Figure 3). Flavonoids comprise a group of polyphenolic substances that are present in most plants. The structural components common to these molecules include two benzene rings on either side of a heterocycle containing three additional carbon atoms. In general, the greater number of hydroxyl substituents on the backbone structure, the stronger the antioxidant activity of the flavonoid. Glycosylation does, however, decrease the antioxidative potential (Miller [1996]). Furthermore, the antioxidative activity in the membrane system depends not only on the number of hydroxyl groups but also on the polarity and hydrophobicity of the tested compounds (Wu et al. [2007]). This study demonstrated that all seven of the isolated compounds from *G. Paraguayense* possessed antioxdative activity. Although those flavonoid compounds showed less antioxidant properties than that of quercetin, our standard reference, it was evident that those compounds may be responsible for the functional ingredients in *G. paraguayense*.

**Table 3:** **50% Inhibition concentrations (IC**_**50**_ **) for radical-scavenging activity and lipid peroxidation inhibition of compounds 1–7 isolated from** ***G. paraguayense***

Compound	***DPPH radical-scavenging activity***(μM)	***ABTS***^***+***^***radical-scavenging activity***(μM)	***Lipid peroxidation inhibition***(μM)
**1**	7.88 ± 0.57	5.69 ± 0.31	10.76 ± 0.10
**2**	144.93 ± 3.56	96.81 ± 2.40	9.10 ± 0.17
**3**	8.40 ± 0.06	9.22 ± 0.67	46.54 ± 1.01
**4**	108.47 ± 6.94	152.21 ± 12.16	17.45 ± 0.18
**5**	11.06 ± 0.34	14.50 ± 1.24	1.67 ± 0.01
**6**	351.89 ± 17.53	277.44 ± 10.01	330.64 ± 12.63
**7**	19.18 ± 0.91	15.28 ± 0.06	2.96 ± 0.06
Quercetin	0.475 ± 0.00	0.35 ± 0.01	0.07 ± 0.00

Values are given as mean ± S.D. (n = 3).

## Conclusions

In this study of the constituents of the leaves of *G. paraguayense* E. Walther, we isolated four major components from its MeOH extract and determined their structures to be (acetylated) HMG-substituted flavonol glycosides, which are rare in nature. Quantitative analysis results indicated that the four major flavonol glycosides totaled 17.3 mg/g of the extract (equivalent to 0.2 mg/g of fresh leaves), a notably high abundance of these special flavonoids in the leaves of this plant. All seven of the isolated compounds possessed antioxdative activity, including radical scavenging, reducing capability and lipid peroxidation inhibitory effect. In future work, due to the similarities of the structures of the four main constituents isolated herein to those commercialized inhibitors as HMG-CoA reductase, further investigation of their bioactivities or pharmacological activities will be continued.

## Authors’ original submitted files for images

Below are the links to the authors’ original submitted files for images. Authors’ original file for figure 1 Authors’ original file for figure 2 Authors’ original file for figure 3
