# Supplementary figures and images for: Chemical characterization and antioxidative activity of four 3-hydroxyl-3-methylglutaroyl (HMG)-substituted flavonoid glycosides from Graptopetalum paraguayense E. Walther
Source: Bot Stud. 2015 Apr 21;56:8. doi: 10.1186/s40529-015-0088-4 (PMC5430333; doi:10.1186/s40529-015-0088-4)

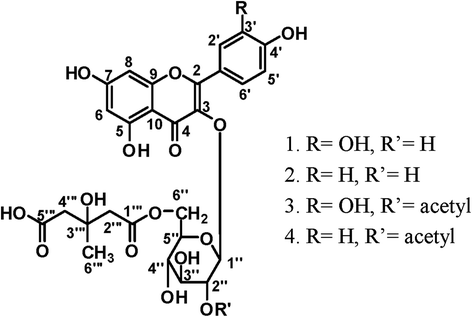

Supplement: Supplementary file 1 — Authors’ original file for figure 1 [file 40529_2015_88_MOESM1_ESM.gif]

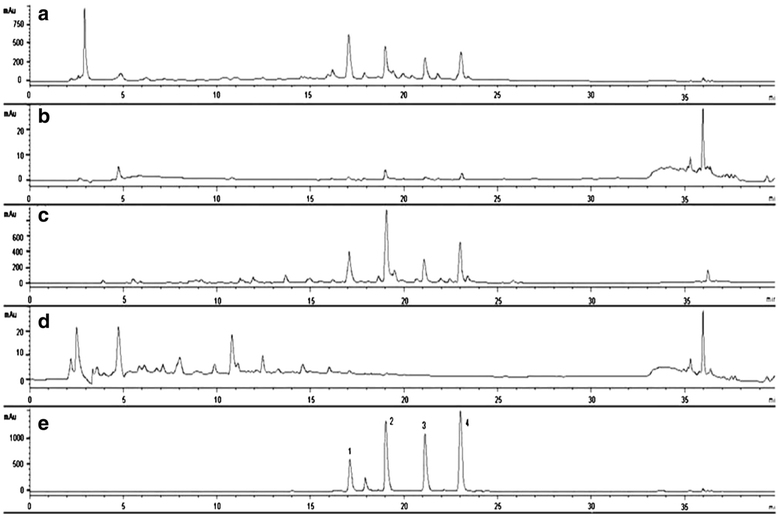

Supplement: Supplementary file 2 — Authors’ original file for figure 2 [file 40529_2015_88_MOESM2_ESM.gif]

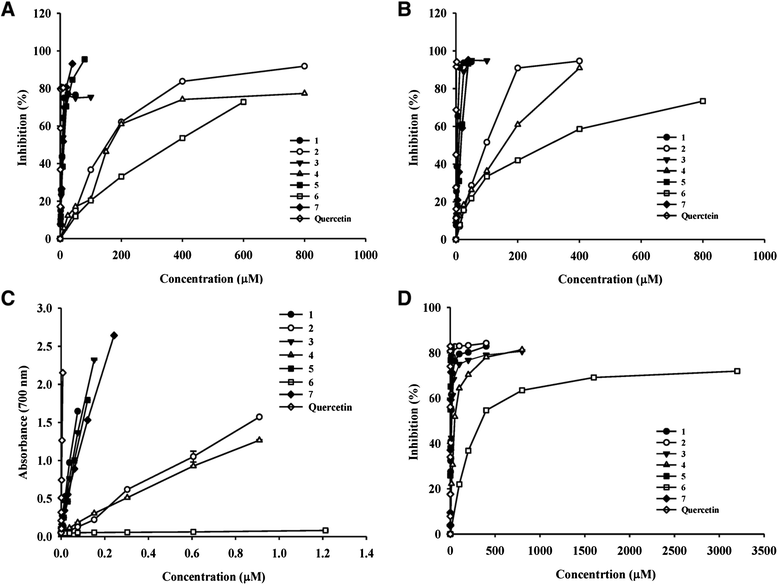

Supplement: Supplementary file 3 — Authors’ original file for figure 3 [file 40529_2015_88_MOESM3_ESM.gif]
